# Supplementary material for: Intimate physical contact between people from different households during the COVID-19 pandemic: a mixed-methods study from a large, quasi-representative survey (Natsal-COVID)
Source: BMJ Open. 2022 Feb 9;12(2):e055284. doi: 10.1136/bmjopen-2021-055284 (PMC8829844; doi:10.1136/bmjopen-2021-055284)
Supplement: Supplementary data [file bmjopen-2021-055284supp001.pdf]

Supplementary Material

Table 1: Proportions, crude, age-adjusted (aOR) and ORs adjusted for age, gender and relationship status (AOR) of reporting intimate physical contact in the past four weeks with a person who lives outside their household (IPCOH) in men and women aged 18-59 years in Britain (n=6654)

Category	% of sample	% reporting IPCOH (95% CI)	Crude OR (95% CI)	aOR (95% CI) ±	AOR (95% CI)	Denominator (unweighted, weighted)
**All participants**	100	9·9 (9·1-10·6)	··	··	··	6654, 6654
**Demographic factors**						
**Age group (years)**			**p<0·0001**	**p<0·0001**	**p<0·0001**	
18-24	13·5	17·7 (15·4-20·3)	3·51 (2·74-4·5)	3·51 (2·74-4·50)	1·94 (1·46-2·60)	1046, 896
25-34	26·4	13·2 (11·6-14·9)	2·47 (1·96-3·11)	2·47 (1·96-3·11)	2·57 (2.00-3·33)	1911, 1753
35-44	24·0	8·0 (6·7-9·5)	1·41 (1·08-1·84)	1·41 (1·08-1·84)	1·65 (1·23-2·22)	1465, 1595
45-59	36·2	5·8 (4·9-6·8)	1·00	1·00	1·00	2232, 2410
**Gender** †			**p=0·024**	**p=0·0088**	**P<0·0001**	
Men	49·8	10·9 (9·8-12·1)	1·00	1·00	1·00	3187, 3310
Women	49·9	8·8 (7·9-9·8)	0·80 (0·67-0·94)	0·80 (0·67-0·94)	0·64 (0·53-0·78)	3443, 3320
**Ethnicity**			**p=0·74**	**p=0·040**	**p=0·044**	
White¶	85·7	10·1 (9·3-10·9)	1·00	1·00	1·00	5837, 5593
Asian/ Asian British¤	8·1	8·6 (6·2-11·8)	0·83 (0·58-1·2)	0·63 (0·44-0·91)	0·62 (0·40-0·98)	395, 530
Black / African / Caribbean / Black British¥	3·4	8·8 (4·8-15·6)	0·90 (0·45-1·65)	0·63 (0·33-1·23)	0·55 (0·25-1·21)	127, 221
Mixed/ multiple ethnic groups /other^	2·8	10·7 (7.0-16·2)	1·10 (0·66-1·74)	0·76 (0·46-1·25)	0·64 (0·36-1·13)	169, 185
**Sexual Identity- self-reported**			**p<0·0001**	**p<0·0001**	**p=0·0027**	
Heterosexual	96	9·6 (8·9-10·4)	1·00	1·00	1·00	5762, 6291
Gay or Lesbian	1·8	19·5 (15·3-24·6)	2·28 (1·68-3·11)	2·50 (1·82-3·45)	1·94 (1·31-2·87)	326,118
Bisexual	1·4	16·9 (13·3-21·1)	1·91 (1·42-2·56)	1·52 (1·12-2·05)	1·50 (1·52-2·25)	393, 93
Other	0·8	14·9 (8·1-25·9)	1·65 (0·82-3·30)	1·28 (0·58-2·81)	1·28 (0·62-2·62)	74, 51

**Region**			**p=0·28**	**p=0·48**	**p=0·97**	
England	86·7	9·6 (8·9-10·4)	1·00	1·00	1·00	5887, 5770
Scotland	8·6	11·7 (9·1-14·9)	1·24 (0·93-1·67)	1·19 (0·89-1·60)	1·03 (0·72-1·47)	509, 572
Wales	4·7	11.0 (7·6-15·5)	1·16 (0·77-1·75)	1·09 (0·72-1·64)	1·05 (0·67-1·62)	258, 312
**Rurality**			**p=0·037**	**p=0·19**	**p=0·10**	
Urban	85·4	10·1 (9·3-11)	1·00	1·00	1·00	4895, 4896
Rural	14·6	7·8 (6·2-9·8)	0·75 (0·57-0·98)	0·83 (0·64 -1·10)	0·76 (0·55-1·05)	846, 840
**Education**			**p=0·68**	**p=0·40**	**p=0·82**	
No qualification	4·3	8·4 (5·6-12·6)	1·00	1·00	1·00	268, 283
Below degree	48·4	10·1 (9·1-11·2)	1·22 (0·77-1·92)	1·22 (0·76-1·95)	1·13 (0·67-1·9)	3195, 3221
Degree or above	47·3	9·8 (8·7-10·9)	1·17 (0·74-1·86)	1·10 (0·68-1·75)	1·17 (0·69-1·97)	3191, 3149
**Social grade**			**p=0·083**	**p=0·089**	**p=0·049**	
A Upper middle class/ B Middle class	22·6	9·7 (8·3-11·3)	1·00	1·00	1·00	1652, 1506
C1 Lower middle class/C2 Skilled working class	52·7	10·2 (9·5-11·7)	1·10 (0·9-1·35)	1·17 (0·95 -1·44)	1·01 (0·79-1·28)	3442, 3508
D Working class/ E Lower level of subsistence	24·7	8·5 (7·2-10.0)	0·86 (0·67-1·1)	0·94 (0·72 -1·21)	0·75 (0·56-1·00)	1560, 1640
**Behavioural factors**						
**Current Relationship Status**			**p<0·0001**	**p<0·0001**	**p<0·0001**	
Steady and living together*	58·6	2·5 (2·1-3·1)	1.00	1·00	1·00	3827, 3889
Steady and not living together*	7·2	56·3 (51·6-60·9)	49·4 (37·2-65·5)	43·9 (32·8-58·8)	46·0 (34·5-61·4)	517, 475
Casual/new**	4·9	36·5 (31·2-42·2)	22·0 (16·0-30·2)	20·7 (15·0-28·4)	20·4 (14·8-27·2)	341, 321
Single	29·4	8·9 (7·6-10·3)	3·72 (2·85-4·86)	3·40 (2·58-4·46)	3·29 (2·50-4·33)	1950, 1947
**Number of sexual partners in the past year§**			**p < 0·001**	**p<0·0001**	**p<0·0001**	
0	30·9	2·2(1·6-3)	0·19(0·14-0·28)	0·18 (0·13-0·26)	0·11 (0·075-0·17)	1663, 1721

1	59·0	10·3(9·3-11·4)	1·00	1·00	1.00	3294, 3288
2	5·5	34·4(29·1-40·2)	4·57(3·47-6·01)	3·94 (2·95-5·27)	2·5 (1·62-3·88)	336, 308
3+	4·7	43·2(37·2-49·3)	6·61(5·02-8·69)	5·60 (4·19-7·46)	5·62 (3·77-8·39)	345, 261
**Condomless sex with a new partner in the past year**[§]			**p<0·0001**	**p<0·0001**	**p<0·0001**	
No	87·9	7·6 (6·9-8·4)	1·00	1·00	1·00	4863, 4861
Yes	12·1	33·3(29·7-37·1)	6·06(4·96-7·4)	5·03 (4·07-6·21)	5·16 (3·87-6·89)	724, 672
**Days drinking alcohol in past week**			**p<0·0001**	**p<0·0001**	**p<0·0001**	
0	37·2	5·8(4·9-6·8)	1·00	1·00	1·00	2407, 2474
1-2	36·3	11·9(10·7-13·3)	2·20(1·78-2·73)	2·16 (1·74-2·68)	2·36 (1·84-3·02)	2466, 2417
3-4	16·6	12·4(10·5-14·6)	2·31(1·79-2·98)	2·32 (1·79-3·01)	2·65 (1·94-3·61)	1118, 1106
5-7	9·9	13·2(10·7-16·2)	2·47(1·84-3·32)	3·00 (2·21-4·06)	3·24 (2·26-4·64)	663, 657
**Alcohol consumption since lockdown**			**p<0·0001**	**p=0·0010**	**p=0·0034**	
Decreased or remained the same	79·2	9·1(8·4-10·0)	1·00	1·00	1·00	5154, 5199
Increased	20·8	13·1(11·3-15·1)	1·50(1·24-1·81)	1·34 (1·14-1·69)	1·43 (1·13-1·83)	1417, 1364
**Health-related factors**						
**General health status**			**p=0·0028**	**p=0·028**	**p=0·0024**	
Good - very good	73·4	10·2(9·4-11·1)	1·00	1·00	1·00	4846, 4870
Fair	21·1	10·1(8·6-11·9)	0·99(0·81-1·21)	1·15 (0·93-1·41)	0·93 (0·73-1·19)	1419, 1400
Bad - very bad	5·6	4·4(2·7-7·1)	0·40(0·24-0·68)	0·54 (0·32-0·93)	0·36 (0·20-0·64)	374, 370
**COVID symptoms and/or diagnosis**			**p<0·0001**	**p=0·0046**	**p=0·019**	
No	81·4	9·1(8·3-9·9)	1·00	1·00	1·00	5358, 5413
Yes	18·6	13·3(11·4-15·4)	1·53(1·26-1·87)	1·34 (1·10-1·65)	1·34 (1·05-1·72)	1289, 1234

**Symptoms of depression (PHQ-2)‡**			**p<0·0001**	**p=0·046**	**p=0·71**	
No	70·9	9·0 (8·2-9·9)	1·00	1·00	1·00	4579, 4642
Yes	29·1	12(10·6-13·6)	1·39(1·16-1·66)	1·20 (1.00-1·45)	1·04 (0·84-1·30)	1964, 1902
**Symptoms of anxiety (GAD-2) ‡**			**p=0·00060**	**p=0·064**	**p=0·51**	
No	71·2	9·06 (8·2-10)	1·00	1·00	1·00	4582, 4679
Yes	28·8	12·0(10·5-13·6)	1·37(1·14-1·63)	1·19 (0·99-1·42)	1·08 (0·86-1·34)	1988, 1889

CI: confidence intervals. OR=odds ratio. AOR = Fully adjusted regressions in which age, gender and relationship status are factored into the model.

PHQ-2=Patient Health Questionnaire (2 item). GAD-2=Generalized anxiety disorder (2 item).

± Age-adjusted ORs, adjusting for age as a continuous variable
†24 participants who identified "in another way" are included in data presented for all participants, but excluded from "Men" and "Women". Trans men and trans women are included in data for men and women, respectively.
¶ White includes all those who identify as White English, Welsh, Scottish, Northern Irish, British, Irish, Gypsy or Irish Traveller, or from any other White background.
¤ Asian includes those who identify as Indian, Pakistani, Bangladeshi, Chinese or from any other Asian background
¥ Black includes those who identify as African, Caribbean, or from any other Black background.
^ Mixed ethnicity includes those who identify as White and Black African, White and Black Caribbean, White and Asian or any other mixed or multiple ethnic background.
§ Includes both opposite-sex and same-sex partners
* Refers to steady, married or civil partnership
** Includes casual, new partner, end of relationship (eg separating), >1 type of partner and "other"
‡ Participants were classified as having symptoms of depression or anxiety if they scored three or more on the patient health questionnaire two item (PHQ-2) or generalised anxiety disorder two item (GAD-2) scales.
All percentages are weighted.
